# Anesthetic Management of a Patient with Advanced Anti-Myelin-Associated Glycoprotein Antibody Neuropathy in the Absence of Measurable Quantitative Neuromuscular Responses: A Case Report

**DOI:** 10.3390/reports9030242

**Published:** 2026-07-27

**Authors:** Jun Yamaguchi, Joho Tokumine, Kiyoshi Moriyama, Harumasa Nakazawa

**Affiliations:** 1 Department of Anesthesiology, Kyorin University School of Medicine, 6-20-2 Sinkawa, Mitaka 181-8611, Tokyo, Japan; jun.cal.daidai.7685@gmail.com (J.Y.); jtokumine@ks.kyorin-u.ac.jp (J.T.); mokiyo@ks.kyorin-u.ac.jp (K.M.); 2 Department of Anesthesiology, Tokyo Medical University, 6-7-1 Nishi-Shinjuku, Shinjuku-ku 160-0023, Tokyo, Japan

**Keywords:** anti–myelin-associated glycoprotein (MAG) antibody polyneuropathy, general anesthesia, COVID-19, neuromuscular blocking agent, neuromuscular monitoring

## Abstract

**Background and Clinical Significance:** Anti–myelin-associated glycoprotein (MAG) antibody polyneuropathy is a rare, chronic IgM-mediated demyelinating peripheral neuropathy predominantly affecting sensory nerves in older adults, commonly in association with monoclonal gammopathy of undetermined significance. Reports describing anesthetic management in patients with this condition remain extremely limited, and no specific guidelines currently exist regarding neuromuscular blocking agent (NMBA) use or neuromuscular monitoring in this population. **Case Presentation:** A 79-year-old man with anti-MAG antibody polyneuropathy (diagnosed in 2007) and IgM monoclonal gammopathy of undetermined significance developed disproportionate progressive lower-extremity weakness and became wheelchair-dependent following COVID-19 infection in 2020. Preoperative evaluation revealed mildly reduced left ventricular function (ejection fraction 49%), mild chronic kidney disease, and marked intrinsic hand muscle atrophy with absent deep tendon reflexes. He was scheduled for robot-assisted radical cystectomy with ileal conduit diversion under combined general and thoracic epidural anesthesia. Before NMBA administration, neuromuscular monitoring was systematically attempted at the ulnar nerve (electromyography and acceleromyography, up to 60 mA/300 μs) and the corrugator supercilii; despite visible muscle contractions following peripheral nerve stimulation, neither modality produced reliable responses at either site. Given the inability to establish reliable monitoring, the administration of NMBAs was considered to carry an unacceptable risk of a prolonged, undetectable blockade. Anesthesia was maintained with deep sevoflurane (2.0–2.5% end-tidal) and remifentanil infusion without NMBAs, titrated to a bispectral index of 40–60. Tracheal intubation was accomplished via video laryngoscopy without NMBA. The 7 h and 30 min surgery was completed without patient movement or surgical compromise. Postoperatively, the patient developed transient upper airway obstruction attributed to glossoptosis, managed successfully with head elevation and nasopharyngeal airway insertion; supplemental oxygen was required until postoperative day 3, and the patient was discharged from the high-dependency unit on postoperative day 5. **Conclusions:** No measurable quantitative neuromuscular response could be obtained in this patient with advanced anti-MAG antibody neuropathy, despite appropriate application of electromyography- and acceleromyography-based monitoring and the presence of visible muscle contractions following peripheral nerve stimulation. In such circumstances, avoiding NMBA administration in favor of deep volatile or intravenous anesthesia with opioid supplementation may represent a reasonable, hypothesis-generating approach in carefully selected patients; this observation does not establish the general superiority of an NMBA-free strategy, and caution is warranted before generalizing it to procedures such as robotic surgery, in which profound neuromuscular blockade is often considered desirable.

## 1. Introduction and Clinical Significance

Anti-myelin-associated glycoprotein (MAG) antibody polyneuropathy is a chronic, slowly progressive demyelinating peripheral neuropathy mediated by IgM monoclonal autoantibodies directed against the MAG epitope. MAG is a glycoprotein expressed predominantly in the periaxonal Schwann cell membrane, where it plays a critical role in axon–glial interaction and myelin compaction. Autoantibody binding to MAG disrupts this interaction and leads to characteristic widening of the myelin lamellae (“widely spaced myelin”), predominantly in the distal portions of peripheral nerves [1]. The disease is almost invariably associated with IgM monoclonal gammopathy, most commonly in the setting of monoclonal gammopathy of undetermined significance (MGUS), and less frequently with Waldenström macroglobulinemia or other low-grade B-cell lymphoproliferative disorders [1,2].

Epidemiologically, anti-MAG neuropathy is predominantly a disease of older adults, with a median age of onset in the sixth to seventh decade and a strong male predominance [1,3]. The estimated prevalence is approximately 1–2 per 100,000 population, though it is likely underdiagnosed given the subtlety of early symptoms [3]. Clinically, patients typically present with slowly progressive distal sensory impairment, gait ataxia due to proprioceptive loss, and action tremor. Weakness, when present, tends to be distal and mild; severe proximal or generalized motor involvement is uncommon and should prompt investigation for additional pathology [1,2]. Electrodiagnostically, the disease produces a characteristic pattern of demyelination with markedly prolonged distal motor and sensory latencies disproportionate to slowing of conduction velocities, a finding termed “distal accentuation” [3].

Treatment of anti-MAG neuropathy remains challenging. Current therapeutic strategies aim to reduce the pathogenic IgM antibody titer through immunotherapy. Rituximab, a monoclonal antibody targeting CD20-positive B cells, is the most widely used agent and has demonstrated modest clinical benefit in randomized trials, although responses are often incomplete and delayed [1,2]. Other approaches include intravenous immunoglobulin (which provides limited benefit in this condition, unlike in chronic inflammatory demyelinating polyneuropathy), plasma exchange, and alkylating agents such as cyclophosphamide or chlorambucil. Despite these treatments, the disease typically follows a slow progressive course, and many patients ultimately develop functional disability including gait impairment and fall risk [1].

From an anesthetic perspective, peripheral neuropathies pose several important challenges. Neuromuscular blocking agents (NMBAs) may exhibit altered pharmacodynamics in patients with peripheral nerve or muscle pathology, and the reliability of standard neuromuscular monitoring may be compromised when nerve conduction is abnormal [4]. The European Neuromuscular Centre (ENMC) consensus statement on anesthesia in patients with neuromuscular disorders emphasizes the importance of individualized NMBA management and vigilant neuromuscular monitoring. However, specific guidance for anti-MAG neuropathy is absent from current recommendations [4]. Because anti-MAG neuropathy is typically considered a sensory-predominant disorder, progressive motor impairment in affected patients may be attributed to the underlying disease without thorough investigation of other potential causes. However, additional factors, including post-infectious neurological deterioration or disuse-related muscle atrophy after severe systemic illness, may contribute to further neurological deterioration and complicate perioperative evaluation.

Reports on anesthetic management in patients with anti-MAG neuropathy remain extremely limited, and no specific recommendations currently exist for NMBA administration or neuromuscular monitoring in this population [4]. Furthermore, the perioperative implications of severe motor dysfunction developing after COVID-19 infection in a patient with pre-existing anti-MAG neuropathy have not been previously described. We report such a patient, in whom reliable neuromuscular monitoring could not be obtained during anesthetic induction despite visible muscle contractions, necessitating an NMBA-free anesthetic strategy. This case highlights the importance of comprehensive preoperative neurological assessment and individualized anesthetic planning in patients with anti-MAG neuropathy, particularly when their clinical course deviates from the expected sensory-predominant phenotype.

## 2. Case Presentation

A 79-year-old man (159 cm, 65 kg) with anti-MAG antibody polyneuropathy was scheduled to undergo robot-assisted radical cystectomy with ileal conduit diversion for bladder cancer. The patient was diagnosed with anti-MAG antibody polyneuropathy and monoclonal gammopathy of undetermined significance (IgM kappa type) in 2007, following several years of progressive distal sensory disturbances including numbness and paresthesia in the hands and feet. Nerve conduction studies performed at diagnosis revealed markedly prolonged distal motor and sensory latencies consistent with distal demyelinating neuropathy. Serum anti-MAG antibody titers were significantly elevated (>10,000 BTU). Intravenous immunoglobulin therapy was attempted but proved ineffective, and cyclophosphamide was subsequently introduced but discontinued because of cardiotoxicity. Since then, the patient was maintained on low-dose prednisolone (5 mg/day) for immunosuppression, with no significant reduction in antibody titers on follow-up. His activities of daily living were fully preserved until 2020, when he was hospitalized for COVID-19 infection (non-severe course not requiring mechanical ventilation). Following discharge, he developed progressive bilateral lower-extremity weakness disproportionate to his prior neurological status. Repeat nerve conduction studies were not performed during this period. Over the subsequent two years, he became progressively unable to walk independently and was wheelchair-dependent by the time of anesthesia consultation. No additional immunotherapy was instituted, as the deterioration was attributed primarily to disease progression in the context of the known anti-MAG neuropathy.

Preoperative evaluation was performed approximately two weeks before surgery. Laboratory investigations showed mild chronic kidney disease (estimated glomerular filtration rate 36.6 mL/min/1.73 m^2^; serum creatinine 1.42 mg/dL), a mildly elevated serum IgM level (780 mg/dL; normal <250 mg/dL), and no coagulopathy (prothrombin time-international normalized ratio 1.02). Hemoglobin was 11.8 g/dL, consistent with mild anemia of chronic disease. Serum albumin was 3.6 g/dL. Spirometry revealed no obstructive or restrictive abnormalities (forced vital capacity 3.12 L [96% predicted]; forced expiratory volume in one second 2.48 L [97% predicted]). Transthoracic echocardiography demonstrated mildly reduced left ventricular systolic function (left ventricular ejection fraction 49%), mild concentric left ventricular hypertrophy, and no significant valvular disease. No wall motion abnormalities were identified. Preoperative neurological evaluation was conducted by the neurology service and focused primarily on the known diagnosis of anti-MAG neuropathy. Physical examination at that time revealed absent deep tendon reflexes throughout all four limbs, bilateral distal sensory loss predominantly affecting vibration and position sense, and bilateral intrinsic hand muscle atrophy with manual muscle testing scores of approximately 4/5 in the thenar muscles and 3/5 in hypothenar and interosseous muscles. Gait assessment was not applicable given the patient’s wheelchair-dependent status. Because anti-MAG neuropathy is generally considered a sensory-predominant disorder and no perioperative management guidelines exist for neuromuscular monitoring or NMBA use in this condition, repeat nerve conduction studies and detailed electrophysiological evaluation were not considered necessary before surgery. Neurological consultation attributed the patient’s functional decline to progression of the underlying anti-MAG neuropathy.

The anesthetic plan was discussed with the surgical team before the day of surgery. Given the patient’s significant motor neuropathy and wheelchair-dependent status, concerns were raised regarding potential sensitivity to NMBAs, difficulty assessing residual neuromuscular blockade, and the risk of postoperative respiratory complications. General anesthesia combined with thoracic epidural analgesia was planned. The epidural catheter was inserted at the T10/11 interspace on the first attempt without complication, and proper placement was confirmed by loss of resistance and a negative aspiration test for blood and cerebrospinal fluid. A test dose of 1.5% lidocaine at 3 mL with epinephrine (1:200,000) was administered, with no signs of intravascular or intrathecal injection. Epidural analgesia was initiated with 0.2% ropivacaine infusion at 4 mL/h after catheter placement was confirmed.

On the day of surgery, standard monitoring was established including five-lead electrocardiography, pulse oximetry, non-invasive blood pressure measurement, and bispectral index (BIS; Covidien, Dublin, Ireland) monitoring. Radial arterial cannulation was performed under local anesthesia for continuous blood pressure monitoring and arterial blood gas analysis. Before induction of general anesthesia, electromyographic (EMG) neuromuscular monitoring electrodes (AF-201P, Nihon Kohden, Tokyo, Japan) were placed on the ulnar side of the right forearm and hand for stimulation at the ulnar nerve and recording at the adductor pollicis. General anesthesia was induced intravenously with remifentanil (0.1 μg/kg/min) and propofol (120 mg). Immediately after loss of consciousness was confirmed (BIS < 60), calibration of the EMG-based train-of-four (TOF) monitor was attempted using the device’s built-in automatic calibration function (Nihon Kohden AF-201P) at a stimulation current of 50 mA and pulse width of 200 μs. Baseline calibration was not attempted before induction of anesthesia, as neuromuscular monitoring was initiated only after loss of consciousness was confirmed. Although slight muscle contraction at the stimulation site was visually apparent, no response was detected by the monitor. We subsequently increased the stimulation current stepwise to 60 mA and widened the pulse width to 300 μs, the maximum settings allowed by the device; electrode repositioning was also performed. Despite these troubleshooting measures, no reliable EMG response was obtained. An acceleromyography-based monitor (TOF-Watch^®^, MSD K.K., Tokyo, Japan) was subsequently applied to the same ulnar nerve site with a piezoelectric sensor at the thumb; however, no measurable responses were obtained at any current setting. After induction, acceleromyography electrodes were applied to the corrugator supercilii, and stimulation was delivered via the facial nerve at the pretragal region. Although four slight muscle twitches were visually observed with each stimulus, the monitor failed to detect them. The entire troubleshooting process, including electrode repositioning and modality switching, took approximately 12 min. To help exclude a purely technical cause of these findings, the acceleromyography-based monitor was independently tested on one of the authors, confirming a normal, reliable response to peripheral nerve stimulation at a control site. The EMG electrodes were single-use and were not retested at a control site; however, visible muscle contraction at the stimulation site was observed with every stimulus, confirming that the stimulus was delivered. The status of the muscle response at the recording site could not be visually confirmed. Taken together, these findings make a device malfunction unlikely and instead suggest a problem with signal conduction within the patient between the stimulation and the recording sites, consistent with the patient’s underlying distal demyelinating neuropathy. Because reliable neuromuscular monitoring could not be ensured at any site using either modality, the anesthesia team determined that NMBA administration carried an unacceptable risk of undetectable, prolonged neuromuscular blockade. Following discussion with the surgical team, it was agreed that robot-assisted laparoscopic surgery could proceed without NMBAs, provided that adequate anesthetic depth was maintained to ensure patient immobility and optimal pneumoperitoneum conditions (intra-abdominal pressure 12 mmHg). Tracheal intubation was performed using video laryngoscopy (McGRATH™ with Blade Size 3, Medtronic Japan Co., Ltd., Tokyo, Japan), which provided an unobstructed glottic view (Cormack–Lehane grade 1) without NMBA. Although transient bucking occurred immediately after the tube passed the vocal cords, it resolved spontaneously within seconds without desaturation, and ventilation was achieved without complication. The cuff was inflated to a pressure of 20 cmH_2_O. Anesthesia was maintained with sevoflurane (end-tidal concentration 2.0–2.5%, approximately 1.0–1.3 MAC) and continuous remifentanil infusion (0.1–0.15 μg/kg/min), titrated to maintain BIS values between 40 and 60. Sevoflurane was selected over total intravenous anesthesia based on institutional practice and familiarity with volatile-based titration to bispectral index targets in this case; in retrospect, given that neuromuscular depression was already a concern in this patient, total intravenous anesthesia may have been preferable, since it would avoid the additional neuromuscular depressant effects of volatile agents and allow more straightforward interpretation of the neuromuscular findings. Epidural ropivacaine infusion (0.2%, 4 mL/h) was continued throughout the procedure to supplement analgesia and reduce inhalational anesthetic requirements. Intraoperatively, BIS remained within the target range (40–55), and the end-tidal sevoflurane concentration was consistently maintained above 2.0%. Throughout the 7 h and 30 min procedure, the patient showed no movement, bucking, or Trendelenburg position-related hemodynamic instability, and the surgical field remained unobstructed during pneumoperitoneum, allowing the surgery to be completed without incident. The operating surgeon did not report any difficulty performing the procedure at any point, and the laparoscopic view remained adequate throughout. Intra-abdominal pressure was maintained at 12 mmHg without need for adjustment, and no additional anesthetic agents beyond those already described were required. The end-tidal sevoflurane concentration used (2.0–2.5%, approximately 1.0–1.3 MAC) was somewhat higher than what would typically be used when NMBAs are administered concurrently, reflecting a deliberate, modest increase in anesthetic depth intended to help ensure immobility in their absence. This level was nonetheless within the standard clinical range and was not associated with any hemodynamically significant effect. Total intraoperative fluid administration was 2100 mL of crystalloid solution, with an estimated blood loss of 320 mL. Vasopressor support (norepinephrine 0.02–0.05 μg/kg/min) was required during the Trendelenburg position phase to maintain adequate mean arterial pressure.

Sevoflurane and remifentanil were discontinued at the end of surgery. The patient regained spontaneous ventilation within 5 min. Full consciousness and the ability to follow verbal commands were confirmed approximately 10 min after discontinuation of anesthetic agents. Before tracheal extubation, neuromuscular function was assessed using standardized clinical criteria, as no measurable quantitative neuromuscular response could be obtained: (1) spontaneous eye opening and sustained ability to follow verbal commands (confirmed by sustained eye opening for ≥5 s and hand squeeze on request); (2) adequate spontaneous ventilation with tidal volume exceeding 6 mL/kg and respiratory rate below 12 breaths/min; (3) ability to perform voluntary movements, including attempted head elevation for ≥5 s and leg elevation; and (4) presence of cough reflex and airway defense reflexes confirmed by laryngeal response to suction. All criteria were satisfied, and the tracheal tube was removed. Immediately after extubation, the patient complained of dyspnea, and oxygen saturation decreased from 100% to 92% on pulse oximetry within approximately 60 s. Glossoptosis was suspected as the primary cause, given the patient’s pre-existing facial and/or oropharyngeal muscle weakness. Supplemental oxygen at 5 L/min via face mask was administered, the head of the bed was elevated to 30 degrees, and a nasopharyngeal airway (size 26 Fr) was inserted. Oxygenation improved promptly to SpO_2_ 98% within 2 min without further intervention. Arterial blood gas analysis at 30 min after extubation showed pH 7.38, PaO_2_ 108 mmHg, PaCO_2_ 42 mmHg, and HCO_3_^−^ 24.8 mEq/L on 3 L/min oxygen via nasal cannula, demonstrating adequate ventilation without CO_2_ retention. The patient was transferred to the high-dependency unit for close monitoring. The nasopharyngeal airway was removed on postoperative day 1 without recurrence of airway obstruction. Supplemental oxygen via nasal cannula was required until postoperative day 3, after which the patient maintained SpO_2_ ≥ 95% on room air. His respiratory condition gradually improved, and he was transferred to the general ward on postoperative day 5 without further complications. The patient’s postoperative analgesic requirements were well managed with the thoracic epidural infusion, and pain scores remained ≤ 3 on a numeric rating scale throughout the hospital stay.

**Figure 1:**
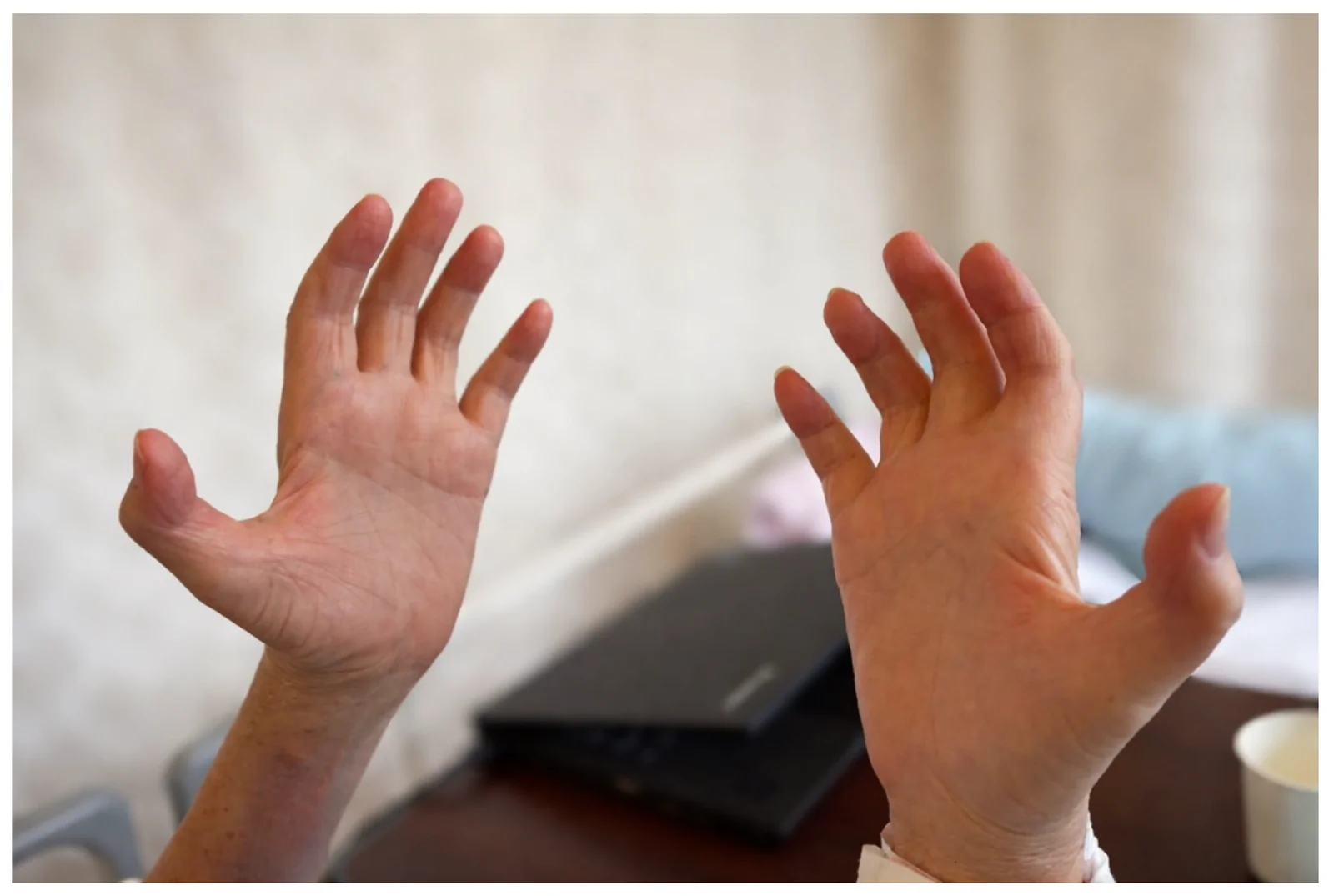
Both hands showing atrophy of the interosseous muscles, particularly the first dorsal interosseous muscle, with depression of the first web space, suggesting involvement of ulnar nerve–innervated intrinsic hand muscles.

Postoperative neurological assessment revealed mild muscle weakness on manual muscle testing, with scores of 4/4 in the thenar muscles, 3/4 in the hypothenar muscles, and 3/4 in the superficial and deep flexors of the ring finger (right/left). Light touch sensation was absent throughout the left lower limb and decreased in the median and ulnar nerve distributions of the right upper limb. Temperature sensation was decreased in the ulnar nerve distribution of the right palm. Vibration and position senses were absent in both lower limbs. Furthermore, both hands showed marked atrophy of the interosseous muscles, particularly the first dorsal interosseous muscle, with a visible depression in the first web space between the thumb and index finger (Figure 1).

## 3. Discussion

This report describes a patient with advanced anti-MAG antibody neuropathy in whom no measurable quantitative neuromuscular response could be obtained despite appropriate application of electromyography- and acceleromyography-based monitoring, prior to any neuromuscular blocking agent (NMBA) administration. The principal clinical lesson of this case is that quantitative neuromuscular monitoring may be unobtainable in patients with advanced anti-MAG antibody neuropathy, and that NMBA administration should be avoided in such circumstances given the resulting inability to detect prolonged or unpredictable neuromuscular blockade. Section 3.1 discusses possible explanations for the motor dysfunction observed in this patient, including progression of anti-MAG neuropathy itself and a masked contributing cause potentially unmasked or exacerbated by COVID-19, without asserting either as established; Section 3.2 and Section 3.3 present postoperative airway findings and the relevant NMBA literature as supporting observations; and Section 3.4 discusses the absence of measurable neuromuscular responses and the rationale for avoiding NMBAs in this specific circumstance. This observation is presented as hypothesis-generating rather than as established evidence for a general NMBA-free anesthetic strategy.

### 3.1. Motor Dysfunction in Anti-MAG Neuropathy

Diseases conventionally regarded as sensory-predominant, such as anti-MAG antibody neuropathy, can nonetheless progress to involve clinically significant motor function; alternatively, such a pre-existing diagnosis may mask a separate, easily overlooked motor process. Both possibilities are considered below. Anti-MAG antibody neuropathy is a chronic demyelinating neuropathy characterized primarily by distal sensory impairment, sensory ataxia, and slow disease progression. Although motor involvement may occur in advanced stages, it is generally less prominent than the sensory manifestations and usually develops gradually over many years [1,2,3]. Given these well-recognized clinical characteristics, the progressive motor dysfunction observed in our patient after COVID-19 infection was initially considered a manifestation of the underlying anti-MAG neuropathy.

However, the severity of motor impairment in this case appeared disproportionate to the typical clinical course of anti-MAG neuropathy alone. After hospitalization for COVID-19, the patient developed progressive lower-extremity weakness and eventually became wheelchair-dependent. Furthermore, the postoperative neurological examination revealed marked intrinsic hand muscle atrophy and clinically significant motor deficits. These findings suggest that mechanisms beyond anti-MAG neuropathy progression may have contributed to the patient’s neurological deterioration.

One possible explanation is that SARS-CoV-2 infection induced immune dysregulation, exacerbating the underlying anti-MAG neuropathy through enhanced IgM-mediated immune responses. Another possibility is the development of a superimposed COVID-19-related immune neuropathy. Various immune-mediated neuropathies, including Guillain–Barré syndrome and other motor-predominant neuropathies, have been reported after SARS-CoV-2 infection [5,6,7,8]. In addition, prolonged hospitalization, reduced physical activity, and subsequent wheelchair dependence after COVID-19 infection may have led to substantial disuse muscle atrophy. The marked atrophy of the intrinsic hand muscles may reflect chronic denervation associated with peripheral neuropathy, although concomitant disuse-related muscle wasting cannot be excluded. Importantly, the pre-existing diagnosis of anti-MAG neuropathy may have led clinicians to attribute the patient’s functional decline primarily to progression of the known neurological disease, thereby masking the potential contributions of COVID-19-related immune neuropathy and disuse muscle atrophy.

In retrospect, this diagnostic assumption likely contributed to insufficient preoperative neurological assessment. Detailed evaluation of motor function and nerve conduction studies were not performed because the patient’s neurological symptoms were considered compatible with progression of his established anti-MAG neuropathy. Consequently, the extent and etiology of his motor dysfunction remained unclear before surgery. We acknowledge that the absence of repeat nerve conduction studies, electromyography, and formal respiratory muscle function testing, both during the two-year interval of progressive decline and preoperatively, represents a genuine limitation in this patient’s clinical management, not merely a limitation of the present report; consequently, the neurological deterioration was incompletely characterized, and this should be borne in mind when interpreting the perioperative findings described below, including the neuromuscular monitoring failure and postoperative respiratory course, and when drawing any clinical conclusions from this case. This case highlights the importance of comprehensive neurological assessment in patients with chronic neuropathies who develop significant functional deterioration after systemic illness, particularly when the clinical course deviates from the typical disease phenotype.

### 3.2. Perioperative Airway Management of Patients with Neuropathies

The perioperative management of patients with clinically relevant motor neuropathies is often challenging because neurological involvement may extend beyond the limbs to include the respiratory, laryngeal, and pharyngeal muscles. Such involvement may increase the risk of perioperative respiratory complications, impaired airway protection, and delayed recovery after general anesthesia. Indeed, our patient developed postoperative dyspnea requiring prolonged supplemental oxygen therapy despite the absence of neuromuscular blocking agent administration. Although glossoptosis was considered the most likely explanation, occult dysfunction of the upper airway or respiratory muscles related to the underlying neuromuscular disorder could not be completely excluded. We present this airway finding as a supporting observation relevant to perioperative planning in this population, rather than as the principal focus of this report. We also consider it important to critically distinguish the intraoperative benefit of avoiding NMBAs from this patient’s overall perioperative course. Despite the NMBA-free strategy, the patient still required nasopharyngeal airway support, supplemental oxygen until postoperative day 3, and admission to the high-dependency unit until postoperative day 5. The findings indicate clinically relevant postoperative respiratory dysfunction. This suggests that avoiding NMBAs did not fully mitigate the risk of postoperative respiratory complications in this patient, and that the observed course more likely reflects the severity of the underlying neuromuscular disease itself than a specific consequence of the anesthetic strategy. Accordingly, we do not attribute the overall favorable outcome of this case solely to the NMBA-free approach.

### 3.3. Neuromuscular Blocking Agents in Demyelinating Neuropathies

As no NMBA was administered in the present case, this section is presented briefly as context for the neuromuscular monitoring findings that are the central focus of this report, rather than as a comprehensive pharmacological review. Previous reports indicate that both NMBA pharmacodynamics and the reliability of neuromuscular monitoring vary considerably across demyelinating and hereditary peripheral neuropathies, ranging from prolonged NMBA sensitivity and delayed recovery in chronic inflammatory demyelinating polyneuropathy and distal acquired demyelinating symmetric neuropathy [9,10] to attenuated or unpredictable NMBA responses in Charcot–Marie–Tooth disease, where EMG-based monitoring has similarly proved unreliable because of underlying nerve conduction abnormalities [11], a scenario that parallels our own case. The mechanisms underlying variable NMBA responses in demyelinating neuropathies likely reflect opposing denervation-related effects: reduced numbers of functional motor units may increase sensitivity to non-depolarizing NMBAs, whereas extrajunctional acetylcholine receptor upregulation from chronic denervation may instead reduce it [12,13]. Most directly relevant to the present report are the predominant distal demyelination and widened periaxonal myelin sheaths characteristic of anti-MAG neuropathy, which would be expected to impair distal motor nerve signal propagation, potentially attenuating both EMG-based and acceleromyographic monitoring responses. In our case, we considered a purely technical cause of the observed monitoring failure unlikely, given that visible muscle contraction confirmed intact stimulus delivery at the stimulation site and that the acceleromyography monitor itself was independently verified on a control site. Of course, the possibility cannot be excluded that the motor nerve dysfunction is attributable to a cause other than anti-MAG neuropathy itself (such as disuse or sequelae of COVID-19 infection); however, since this finding is not inconsistent with a conduction disturbance due to distal demyelination, it suggests that standard neuromuscular monitoring may be unreliable even before NMBA administration in patients with advanced anti-MAG neuropathy.

### 3.4. Absence of Measurable Neuromuscular Responses and the Rationale for Avoiding NMBAs

In the present case, placement of neuromuscular monitoring before NMBA administration proved clinically valuable. Despite visible muscle contractions following peripheral nerve stimulation, neither electromyography- nor acceleromyography-based monitoring produced reliable responses. Although detailed neurological investigations had not been performed preoperatively, this unexpected finding served as an important warning sign that the patient’s neuromuscular dysfunction had been underestimated. Had NMBAs been administered routinely, prolonged paralysis, residual postoperative weakness, or unpredictable responses might have occurred without any objective means of assessment. Because reliable neuromuscular monitoring could not be established, we considered administration of NMBAs to carry an unacceptable level of risk and elected to maintain surgical immobility using deep anesthesia and opioid analgesia alone. This strategy enabled successful robotic surgery without patient movement or surgical interference. We acknowledge that failure of neuromuscular monitoring alone does not represent an established contraindication to NMBA administration: anti-MAG neuropathy itself has not been shown to cause clinically significant hypersensitivity to non-depolarizing NMBAs or prolonged neuromuscular blockade, and sugammadex was available for reversal if required. However, our decision was informed by evidence from other demyelinating peripheral neuropathies indicating that these reassurances do not always hold. In a patient with undiagnosed Charcot–Marie–Tooth disease type 1A, an induction dose of rocuronium produced a neuromuscular respiratory paralysis refractory to a cumulative sugammadex dose of 1200 mg (17.3 mg/kg)—well above doses shown to reverse blockade in patients without neuromuscular disease—with recovery requiring 18 h of mechanical ventilation [14]. This case illustrates that sugammadex availability does not guarantee timely or complete reversal in patients with demyelinating peripheral neuropathies, and that the extent of underlying motor nerve involvement may be substantially underestimated preoperatively, as was the case in this patient. Our own patient’s diagnosis of anti-MAG neuropathy, a disease conventionally regarded as sensory-predominant, may similarly have masked a greater degree of motor nerve involvement than was clinically apparent preoperatively, as discussed in Section 3.1. Given this precedent for reversal-resistant, prolonged paralysis in a related demyelinating neuropathy, and our inability to reliably monitor neuromuscular function in this patient, we judged that even a low probability of a similarly refractory response represented an unacceptable risk in the absence of validated means to detect it. We recognize that avoiding NMBAs poses its own challenges for robotic surgery, which ordinarily benefits from profound neuromuscular blockades; however, once this finding was encountered, we judged the risk of prolonged blockades to outweigh these challenges. We present this decision as one reasonable option for managing this specific, unanticipated intraoperative situation, rather than as a general recommendation for anti-MAG neuropathy or for demyelinating neuropathies more broadly; it reflects an individualized, precautionary clinical judgment made under diagnostic uncertainty, rather than an evidence-based management standard.

Another limitation of our anesthetic management was the use of sevoflurane for maintenance of anesthesia. Volatile anesthetics are known to potentiate neuromuscular blockade and may independently depress neuromuscular transmission [15]. In a patient with suspected neuromuscular dysfunction and no measurable quantitative neuromuscular response, total intravenous anesthesia would have been preferable because it minimizes additional pharmacological effects on neuromuscular transmission and facilitates more accurate interpretation of neuromuscular function [16]. Although no intraoperative complications occurred, the use of volatile anesthesia may have further complicated assessment of neuromuscular status and should be regarded as a limitation of our management strategy.

### 3.5. Practical Recommendations for Anesthetic Management

Rather than emphasizing the uniqueness of this single case, we focus here on its practical clinical implications: quantitative neuromuscular monitoring may be unobtainable even before NMBA administration in patients with advanced anti-MAG antibody neuropathy, and this finding, rather than the neuropathy diagnosis alone, may reasonably inform a decision to avoid NMBAs. This case suggests that satisfactory surgical conditions may be achievable without NMBA administration in carefully selected patients with advanced anti-MAG antibody neuropathy. However, we present this observation as hypothesis-generating rather than as evidence supporting a general NMBA-free anesthetic strategy; the omission of NMBAs is itself an established approach in selected neuromuscular disorders, and further studies are required to determine the safety and applicability of this approach in similar patients. This case demonstrates that patients with anti-MAG neuropathy may develop clinically significant motor dysfunction that is not fully explained by the typical sensory-predominant phenotype of the disease. Additional contributing factors, including neurological complications following SARS-CoV-2 infection or disuse-related muscle atrophy, should be considered when functional decline appears disproportionate to the expected disease course. Although the precise contribution of COVID-19-related immune neuropathy versus disuse atrophy in our patient could not be definitively established without repeat electrodiagnostic studies, the clinical trajectory and disproportionate motor involvement suggest that both mechanisms may have contributed.

Based on our experience with this case, we propose several practical recommendations for the anesthetic management of patients with anti-MAG neuropathy or similar chronic demyelinating neuropathies. First, comprehensive preoperative neurological assessment, including updated nerve conduction studies and quantitative motor function testing, should be considered when the degree of motor impairment appears disproportionate to the typical disease phenotype or when functional decline has accelerated following systemic illness. Second, neuromuscular monitoring should be attempted before any NMBA administration to assess baseline reliability; the failure to obtain reliable monitoring signals should itself be treated as a clinically significant finding that informs the subsequent anesthetic plan. Third, when neuromuscular monitoring cannot be established, an NMBA-free strategy using deep inhalational or intravenous anesthesia with opioid supplementation may represent a viable and safe alternative for procedures where absolute muscle relaxation is not required. Fourth, total intravenous anesthesia is preferable to volatile anesthesia in this setting, as it avoids the additional neuromuscular depressant effects of volatile agents and allows more straightforward clinical assessment of neuromuscular recovery. Finally, given the risk of postoperative upper airway dysfunction in patients with oropharyngeal muscle involvement, rigorous extubation criteria and the availability of airway adjuncts such as nasopharyngeal airways should be ensured before tracheal extubation.

### 3.6. Limitations

This report has several limitations. First, it describes a single case, and our observations are therefore hypothesis-generating rather than evidence-based. Second, the findings cannot be generalized to all patients with anti-MAG antibody neuropathy, given the variability in disease severity and phenotype in this population. Third, the use of sevoflurane, known to potentiate neuromuscular blockade, may have contributed to the observed muscle relaxation, and this cannot be distinguished from the effect of the underlying neuropathy. Finally, this case does not establish the superiority or general applicability of an NMBA-free anesthetic strategy; it illustrates one approach to a specific, unanticipated intraoperative situation, and further studies are required before broader recommendations can be made.

## 4. Conclusions

The principal clinical lesson of this report is that quantitative neuromuscular monitoring may be unobtainable in patients with advanced anti-MAG antibody neuropathy—a disease conventionally regarded as sensory-predominant—despite appropriate application of electromyography—and acceleromyography-based devices, as well as that NMBA administration should be avoided in such circumstances given the resulting inability to detect prolonged or unpredictable neuromuscular blockade. In this patient, the underlying motor dysfunction may have reflected progression of anti-MAG neuropathy itself, or another underlying cause masked by the pre-existing diagnosis, potentially unmasked or exacerbated by COVID-19. Comprehensive preoperative neurological assessment may help identify such patients, particularly when functional decline appears disproportionate to the expected disease course. This case suggests that satisfactory surgical conditions may be achievable without NMBA administration when quantitative neuromuscular monitoring is unavailable; however, this observation is hypothesis-generating rather than evidence establishing the general safety or applicability of an NMBA-free strategy, and further studies in similar patients are required before broader recommendations can be made.

## Data Availability

The original data presented in the study are included in the article, further inquiries can be directed to the corresponding author.

## Abbreviations

The following abbreviations are used in this manuscript:

MAG	Myelin-associated glycoprotein
MGUS	Monoclonal gammopathy of undetermined significance
NMBA	Neuromuscular blocking agent
EMG	Electromyography
TOF	Train-of-four
COVID-19	Coronavirus disease 2019
SARS-CoV-2	Severe acute respiratory syndrome coronavirus 2
IgM	Immunoglobulin M
